# Early health economic evaluation of the future potential of next generation artificial vision systems for treating blindness in Germany

**DOI:** 10.1186/s13561-014-0027-1

**Published:** 2014-11-01

**Authors:** Bjoern Schwander

**Affiliations:** AHEAD GmbH, Agency of Health Economic Assessment and Dissemination, Arndtstr. 19, 79539 Loerrach, Germany

**Keywords:** Cost-effectiveness, Early economic evaluation, Germany, Retinitis pigmentosa, Blindness, Medical devices, Artificial vision, Markov model, Health care payer perspective

## Abstract

The next generation of artificial vision devices (AVDs), which is currently developed in pre-clinical settings, has the potential to improve the vision of blind patients with retinitis pigmentosa (RP) in a manner that they will be categorized as visual impaired but no more as blind. This unprecedented vision improvement will result in a mentionable quality of life gain which poses the question at which costs the next generation AVDs are to be regarded as cost-effective, from a German healthcare payer perspective. In order to answer this research question a Markov model was developed to simulate and to compare the costs and effects of next generation AVDs versus best supportive care (BSC). Applying the base case settings resulted in incremental costs of €107,925, in 2.03 incremental quality-adjusted life years (QALYs) and in a cost-effectiveness ratio of €53,165 per QALY gained. Probabilistic and deterministic sensitivity analyses as well as scenario analyses for the effect size and the AVD costs were performed in order to investigate the robustness of results. In these scenario analyses a strong variation of the cost-effectiveness results was obtained ranging from €23,512 (best case) to €176,958 (worst case) per QALY gained by AVD therapy. This early health economic evaluation has to handle with three main uncertainty factors: the effect size of next generation AVDs, the costs of next generation AVDs and the WTP threshold that might be applied in RP patients, which reflect the main limitations of the presented assessment. In conclusion the presented early cost-effectiveness evaluation has obtained that next generation AVDs have the potential to be a cost-effective therapy option in patients with RP in Germany. The innovative nature, the high unmet medical need and the expected unprecedented efficacy of next generation AVDs will highly likely lead to the case that even relatively high incremental cost-effectiveness ratios, that have been obtained when simulating various effect and pricing scenarios, will be regarded as acceptable from a German healthcare payer perspective.

## Background

After almost half a century of research activities artificial vision systems are moving in the clinical practice. These devices are designed to provide prosthetic vision to the blind by stimulating localized neural populations in one of the retinotopically organized structures of the visual pathway – typically the retina or visual cortex [1].

According to the World Health Organization (WHO) blindness affects almost 45 million people worldwide (≈300,000 in Germany [2]) and its prevalence constantly increases along with population aging [3]. For some of these diseases, there is currently no efficient treatment for preventing severe visual loss or blindness. This is the case for retinitis pigmentosa (RP) accounting for ≈ 1 million patients worldwide [4] and for ≈ 20,000 patients in Germany [5]. In RP, photoreceptor degeneration leads to a progressive reduction of the visual field often declining to legal blindness; in these patients prosthetic vision is so far the only effective treatment strategy [4].

The current generation of these artificial vision devices (AVDs) provides a level of prosthetic vision that allows RP patients to perceive light, recognize shapes and objects and even read large font print, which is already a substantial progress for the patients. However the visual acuity remains still in a region that is defined as legal blindness (best result 20/1260; legal blindness <20/200) [4,6,7].

Technological advancements, coupled with recent scientific investigations, have transformed the focus of the field from that of whether it is possible to create visual sensations through electrical stimulation to the more important question of how to optimize the perceptions for maximum benefit [8]. The next generation of AVDs, which is currently developed in pre-clinical settings, has the future potential to improve the vision of blind patients in a manner that they will be categorized as visual impaired but no more as blind (visual acuity >20/200) [4]. This unprecedented vision improvement will result in a mentionable quality of life gain which poses the question at which costs the next generation AVDs are to be regarded as cost-effective.

Accordingly the objective of the presented manuscript is to determine at which effect size and at which cost next generation AVDs will be regarded as cost-effective when compared to the current practice (best supportive care) for treating blind patients with RP from a German healthcare payer perspective.

## Methods

A Markov model with yearly cycles consisting of the three health states ‘Blind’, ‘Visual Impaired’ and ‘Death’ was developed in Excel 2010 in order to simulate and to compare the costs and effects of next generation AVDs versus best supportive care (BSC). As there is currently no cure for RP [5], BSC, consisting of all available medical support for patients with RP (e.g. RP related physician visits and rehabilitation, mobility training, vision-enhancing equipment, visual aids etc.), was selected as comparator.

### Health state transition

As the development of these next generation AVDs is currently in a pre-clinical stage, the actual treatment effect is not yet predictable and hence there is a high uncertainty of treatment outcomes; therefore five different AVD responder rate scenarios (proportion of patients that obtain a vision improvement from ‘Blind’ to ‘Visual Impaired’: 25%, 50%, 75%, 100% and 62.5% as average base case) were applied in order to simulate the transition from the health states ‘Blind’ to ‘Visual Impaired’. It was assumed that the responder rate of next generation AVDs will be constant over time – hence it was simulated that patients (responders) that have entered the ‘Visual Impaired’ state will either stay in this state or will die. In case that the patients are treated with BSC it was assumed that the vision cannot be restored (improved) as blindness is currently a chronic (incurable) condition for patients with advanced RP; hence patients treated with BSC cannot move from ‘Blind’ to ‘Visual Impaired’. As there is currently no AVD long-term (end-of-life) data available, not even for the first generation AVDs, the transition from ‘Blind’ or ‘Visual’ Impaired’ to ‘Death’ was simulated on the basis of German life tables [9], irrespective of the therapy approach (AVD or BSC). For the survival analyses a starting age of 51 years (SD 5.3 years) was simulated as this is the mean age for the onset of bilateral blindness in RP [10].

**Figure 1:**
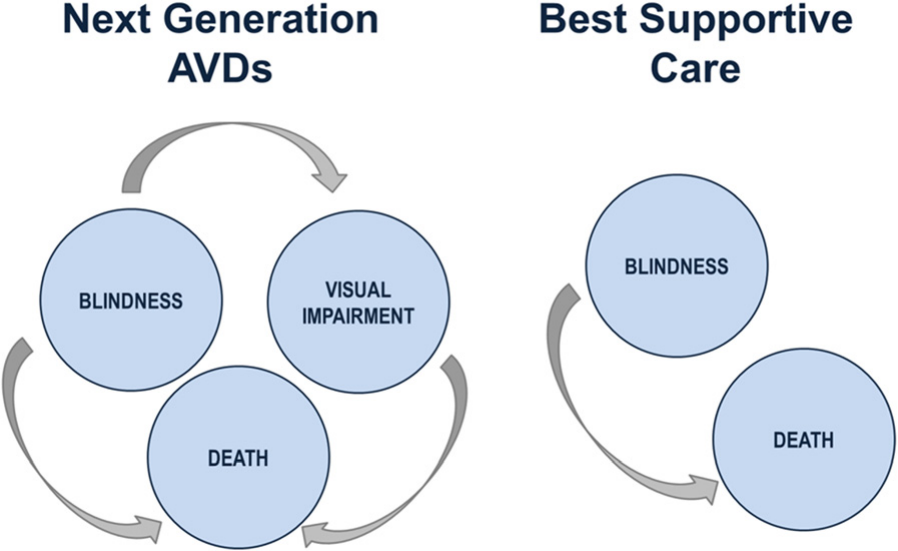
**Overview of Markov health states and of the possible state transitions for AVDs and BSC.**

The Markov health states (illustrated as bubbles) and the possible state transitions (illustrated as arrows) for the AVDs and BSC therapy approaches are shown in Figure 1.

### Cost measures

For the cost estimates a German healthcare payer perspective was applied. Yearly healthcare costs for the health states ‘Blind’ and ‘Visual Impaired’ were determined on the basis of published literature [11,12]. According to Gruess et al. the annual direct vision related healthcare costs are €3,163 for ‘Blind’ patients and €2,845 for ‘Visual Impaired’ patients [11]. These costs related to the reference year 2005 were inflated to 2014 values by applying an average yearly inflation rate of 2,9% (based on the average annual healthcare cost increase of the German statutory health insurance from 2002 to 2012) [12]. Hence the annual costs in 2014 prices were estimated at €4,091 for ‘Blind’ patients and at €3,680 for ‘Visual Impaired’ patients. The costs for the health state ‘Death’ were set to zero. All these annual health state costs were applied for both strategies investigated (AVD and BSC) as long as a patient stayed in the related health state.

As the costs for the next generation AVDs are not yet determined (released) different costing scenarios were assumed using the cost of the currently available first-generation device (Argus II® device) as basis [13]. These costs are currently ranging from €70,000 to €100,000 in the first year (which includes the costs for the device, the implantation, rehabilitation and training) and the annual follow-up costs are estimated at €1,500 in the subsequent years (ongoing training and possible device updates). As the costs for the next generation AVDs could be lower (e.g. due to a more competitive environment) or higher (due to the expected improvement in efficacy) different AVD costing scenarios were analyzed: the first year cost were set at €55,000, €70,000, €85,000 (mean base case), €100,000 and at €115,000 whereas the annual follow-up costs were kept constant at €1,500.

For the BSC strategy it was assumed that the health state costs for ‘Blind’ and ‘Visual Impaired’, determined on the basis of published literature, already include all relevant healthcare payer costs.

### Effect measures

The effect estimates are based on published health related quality-of-life estimates for the health states ‘Blind’ and ‘Visual Impaired’. According to a recent NORC report [14] the average health utility is 0.61 for the health state ‘Blind’ (based on 6 publications with the following utility values: 0.39, 0.51, 0.55, 0.60, 0.79 and 0.80), and 0.77 for ‘Visual Impaired’ (based on 8 publications with the following utility values: 0.62, 0.65, 0.73, 0.76, 0.78, 0.79, 0.87 and 0.96); a health utility of zero was applied for the state ‘Death’.

### Discounting

Costs and effects were discounted by 3% per annum according to German health economic recommendations (Institute for Quality and Efficiency in Healthcare 2009 [15]).

### Sensitivity analyses

Probabilistic sensitivity analyses (by defining reasonable input value distributions and running Monte-Carlo simulations) and deterministic sensitivity analyses (by varying the input value point estimates of the base case to reasonable extreme values) were performed in order to investigate the robustness of results. For both the cost and the effect inputs a normal distribution was estimated by applying a standard deviation of 10% of the mean input value. For the AVD responder rate as well as for first year AVD costs different scenario analyses were performed. A base case that is defined by the mean value of the assumed scenarios for the AVD responder rate and the first year AVD costs was simulated in order to build the basis for performing the deterministic sensitivity analyses. An overview of the input values and the applied distributions for running the probabilistic sensitivity analysis are shown in Table 1.

**Table 1:** **Overview of cost and effect input data applied in the model**

**Parameter**	**Mean input value**	**Standard deviation**	**Distribution**
Age at Therapy Start	51.1	5.3	Normal
Health State Costs ‘Visual Impaired’	€3,680	€368	Normal
Health State Costs ‘Blind’	€4,091	€409	Normal
Health State Costs ‘Death’	€0	€0	None
AVD Costs, first year (Base Case)	€85,000	€8,500	Normal
AVD Costs, first year (Scenario 1)	€55,000	€5,500	Normal
AVD Costs, first year (Scenario 2)	€70,000	€7,000	Normal
AVD Costs, first year (Scenario 3)	€100,000	€10,000	Normal
AVD Costs, first year (Scenario 4)	€115,000	€11,500	Normal
AVD Costs, annual follow-up	€1,500	€150	Normal
Health State Utility ‘Visual Impaired’	0.77	0,077	Normal
Health State Utility ‘Blind’	0.61	0.061	Normal
Health State Utility ‘Death’	0	0	None
Responder Rate AVD (Base Case)	62.5%	None	None
Responder Rate AVD (Scenario 1)	25%	None	None
Responder Rate AVD (Scenario 2)	50%	None	None
Responder Rate AVD (Scenario 3)	75%	None	None
Responder Rate AVD (Scenario 4)	100%	None	None
Discounting of Costs	3%	None	None
Discounting of Effects	3%	None	None

### Model simulations, model outcome and time horizon

The model simulations are based on a Monte-Carlo simulation process that uses 1,000 iterations (random number generator scenarios) in order to investigate the variance of results according to the applied input data distributions. The results are provided as incremental cost-effectiveness ratio that is expressed as the cost per quality-adjusted life year (QALY) gained comparing next generation AVDs versus BSC from a German healthcare payer perspective.

### Willigness-to-pay

As there is no official willingness-to-pay (WTP) threshold for the cost per QALY gained in Germany the results of the assessment were compared to other German cost per QALY outcomes obtained in the field of visual interventions using an overview provided Neubauer et al. 2010 [16] as basis. Furthermore the probability of whether AVDs are a cost-effective at different WTP thresholds was investigated by applying a cost-acceptability analysis.

## Results

### Base case analyses results

Comparing next generation AVDs versus BSC, while applying the base case settings (as described in Table 1), resulted in mean incremental costs of €107,925, in 2.03 mean incremental QALYs and in a cost-effectiveness ratio of €53,165 per QALY gained. The detailed results for both therapy options, including the standard deviation and the confidence intervals (based on the 1,000 iterations), are provided in Table 2 below.

**Table 2:** **Overview of the base case results comparing AVD versus BSC**

**Costs in Euro**	**AVD**	**BSC**	**Incremental**
Mean Costs in Euro	€189,887	€81,962	€107,925
Standard Deviation	€16,732	€12,138	€15,035
2.5% Confidence Interval	€155,538	€57,298	€77,379
97.5% Confidence Interval	€221,407	€106,697	€136,992
**Effect in QALYs**	**AVD**	**BSC**	**Incremental**
Mean QALYs	14.19	12.16	2.03
Standard Deviation	2.12	1.80	1.94
2.5% Confidence Interval	9.86	8.54	−1.70
97.5% Confidence Interval	18.48	15.66	5.87
**Cost-Effectiveness**	**Cost per QALY gained (AVD versus BSC)**		
Cost per QALY gained in Euro	€53,165		
Standard Deviation	€13,273,467		
2.5% Confidence Interval	-€501,604		
97.5% Confidence Interval	€757,628		

*AVD* Artificial Vision Device, *BSC* Best Supportive Care, *QALY* Quality Adjusted Life Year.

### Probabilistic sensitivity analyses results

**Figure 2:**
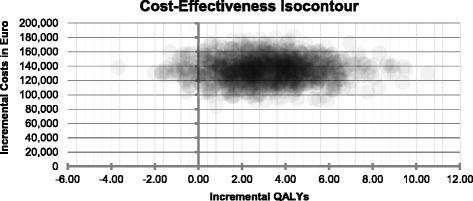
**Base case scatterplot/isocontour comparing AVD versus BSC.**

The fact that the simulations are based on a Monte-Carlo simulation process (that uses 1,000 random number generator scenarios) enables to investigate the possible spread of incremental cost-effectiveness ratio (ICER) estimates when comparing AVD versus BSC. This result deviation is visualized (for the base case setting) in form of an ICER plane that shows the incremental effect (of comparing AVD vs BSC) in QALYs on the x-axis and the incremental costs in euros on the y-axis. This ICER plane is illustrated as a cost-effectiveness scatterplot/isocontour in Figure 2. As it can been seen in this figure most ICER estimates (darkest areas) are located in the upper right corner of the ICER plane which means that AVD is more effective but also more costly compared to BSC and in this case the healthcare payers’ WTP for an additional QALY determines whether AVD will be regarded as cost-effective or not.

### Deterministic sensitivity analyses

**Figure 3:**
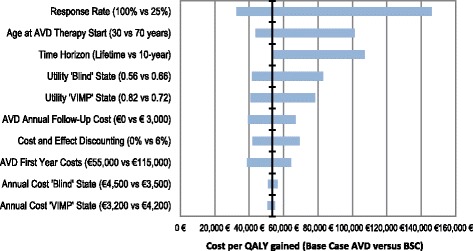
**Base case tornado diagram comparing AVD versus BSC.** Comment: ‘VIMP’ = ‘Visual Impaired’ State.

The influence of single input parameters on the cost-effectiveness analyses results was investigated by applying one-way sensitivity analyses on all relevant input parameters. The results of this deterministic sensitivity analyses are provided for the base case setting as Tornado diagram in Figure 3.

According to the Tornado diagram the variation of the response rate (that will be further investigated in the scenario analyses presented in the next subchapter) showed the strongest impact on the cost-effectiveness analyses results followed by the variation of the age at therapy start and variation of the observation time horizon. Furthermore the variation of the health utility values for the health states ‘Blind’ and ‘Visual Impaired’ and the variation of the annual follow-up costs of AVD therapy were determined as parameters with a strong influence on the results, followed by cost and effect discounting and by the costs of AVD in the first year (which will also be part of the scenario analyses presented in the following). The possible impact of these deterministic sensitivity analyses results on future health economic evaluations will be outlined in the discussion part.

### Responder rate and AVD cost scenario analyses

The outcomes of the scenario analyses, on the AVD responder rate (RR) and on the first year AVD costs, are presented in Table 3. In these analyses a strong variation of the cost-effectiveness results was obtained ranging from €23,512 (best case; RR 100%; AVD first year costs €55,000) to €176,958 (worst case, RR 25%; €115,000) per QALY gained by AVD therapy.

**Table 3:** **Overview of the scenario analyses results**

**Outcome**	**RR 25%**	**RR 50%**	**RR 62.5%**	**RR 75%**	**RR 100%**	**Scenarios**
IC (Euro)	81,640	78,606	77,674	76,658	75,237	AVD Cost €55,000
IE (QALYs)	0.80	1.59	2.03	2.41	3.20	
ICER	**102,050**	**49,438**	**38,263**	**31,808**	**23,512**	
IC (Euro)	96,276	94,752	92,826	91,983	90,158	AVD Cost €70,000
IE (QALYs)	0.80	1.59	2.03	2.41	3.20	
ICER	**120,345**	**59,592**	**45,727**	**38,167**	**28,174**	
IC (Euro)	110,984	109,218	107,925	106,731	104,685	AVD Cost €85,000
IE (QALYs)	0.80	1.59	2.03	2.41	3.20	
ICER	**138,730**	**68,691**	**53,165**	**44,287**	**32,714**	
IC (Euro)	126,384	123,346	122,218	121,913	119,396	AVD Cost €100,000
IE (QALYs)	0.80	1.59	2.03	2.41	3.20	
ICER	**157,980**	**77,576**	**60,206**	**50,586**	**37,311**	
IC (Euro)	141,566	139,301	138,746	137,416	134,948	AVD Cost €115,000
IE (QALYs)	0.80	1.59	2.03	2.41	3.20	
ICER	**176,958**	**87,611**	**68,348**	**57,019**	**42,171**	

*RR* Responder Rate, *IC* Incremental Costs in Euro, *IE* Incremental Effect in QALYs, *ICER* Incremental Cost-Effectiveness Ratio (Cost per QALY gained AVD vs BSC).

### Interpretation of results

In Germany there is no official WTP threshold per QALY gained in order to guide reimbursement decisions. However, systematic comparisons of the cost-effectiveness outcomes of a new intervention to the cost-effectiveness outcomes of already reimbursed intervention(s), which are applied in the same disease area, are usually performed [15]. Such systematic comparisons are usually based on the efficacy frontier approach proposed by the German Institute for Quality and Efficacy in Healthcare (IQWiG) [17]. However for applying this efficacy frontier approach there are specific preconditions to be fulfilled by the cost-effectiveness assessments used as basis: they need to be performed in the same country-setting and in the same disease area, need to use that same efficacy outcome and costing perspective, need to use a comparable comparator, need to use a comparable treatment period and a comparable observation period [18]. For RP there is so far no German cost-effectiveness study published but there are studies published for age-related macular degeneration (ARMD), hence there are at least studies available that are related to the field of visual impairment/blindness.

Neubauer et al. investigated the cost-effectiveness of ranibizumab compared to BSC [placebo; sham injection] (in ARMD from a German healthcare payer perspective), which resulted in costs per QALY gained ranging from €13,505 (4 injections per year) to €87,862 (12 injections per year – as applied in the underlying phase III trials) [16]. In this paper further ARMD cost per QALY gained thresholds are provided whereas photodynamic therapy compared to BSC ranged from €25,193 [19] to €46,535 [20] and pegaptanib versus BSC resulted in €54,252 [21] per QALY gained.

These studies described above used a 2-year treatment time horizon and an observation period of 10 years. Hence beside the fact that these assessments are not performed within the same disease area the different treatment time and observation period do not allow an application of the efficacy frontier approach. Thus it is currently not predictable which WTP thresholds German authorities might apply for AVDs in RP. However considering these cost-effectiveness ratios for ARMD therapies and considering the high unmet medical need in blind RP patients (there is currently no effective therapy) it is highly likely that a cost-per QALY gained threshold of up to €80,000 will be considered as acceptable, as it is the case for ranibizumab in ARMD. As this threshold cannot be verified, different WTP thresholds (€40,000; €60,000 and €80,000) have been applied in order to investigate the probability of whether AVD is a cost-effective treatment strategy in RP when compared to BSC.

**Figure 4:**
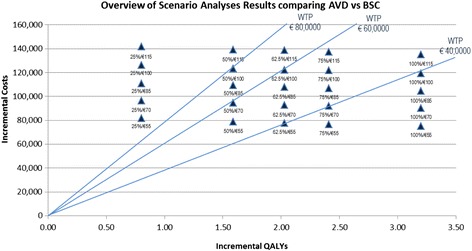
**Scenario analyses results in relation to different WTP thresholds** ***.*** Scenario definition (labels): The percentage defines the responder rate and the Euro value defines the first year AVD costs in thousands (e.g. 25%/€55 means AVD response rate of 25% and the first year AVD costs of €55,000).

In order to enable an interpretation of the various cost-effectiveness outcomes of AMD versus BSC the scenario analyses outcomes presented previously were visualized in Figure 4 in relation to different WTP thresholds.

Above it can be seen that 28% of the scenarios (7 of 25) are below a WTP threshold (per QALY gained) of €40,000; that 60% of the scenarios (15 of 25) are below a WTP threshold of €60,000 and that 76% of the scenarios (19/25) are below a WTP threshold of €80,000.

Additionally three scenarios (best case, base case and worst case) were selected in order to investigate the probability that AVDs (compared to BSC in RP) are a cost-effective intervention in case of assuming different WTP thresholds. This cost acceptability analyses are performed on the basis of the probabilistic sensitivity analyses results (1,000 iterations) that are based on a Monte-Carlo simulation process.

**Figure 5:**
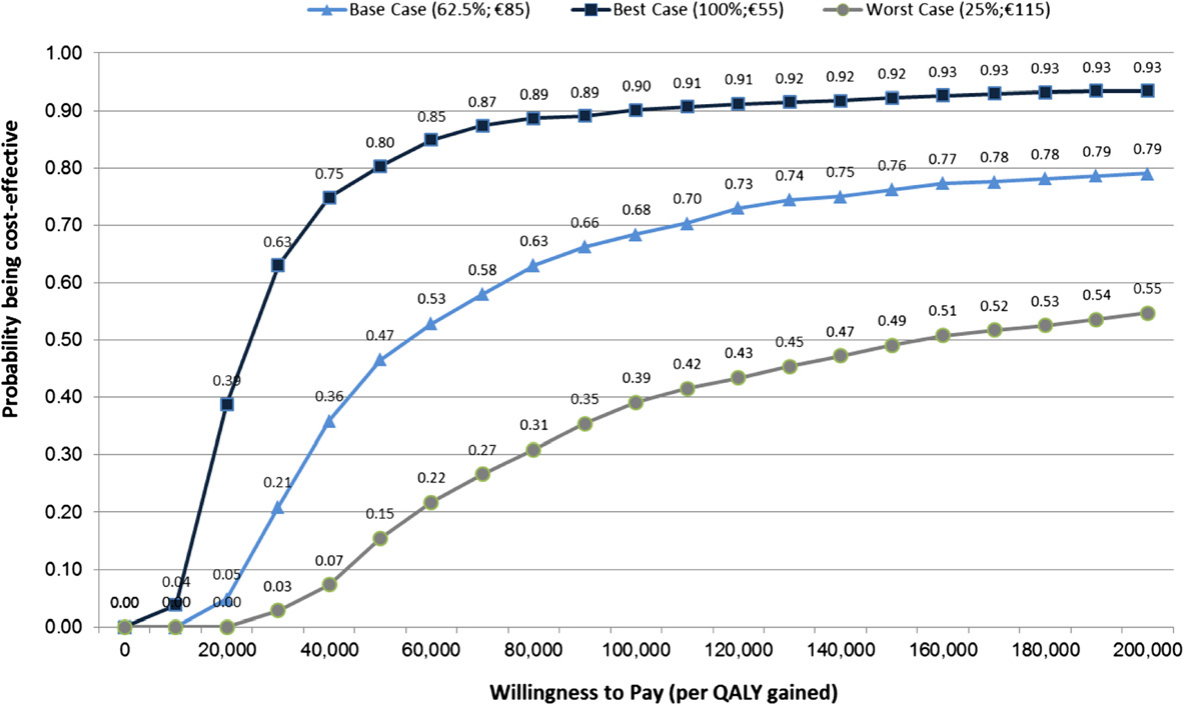
**Cost acceptability curves comparing AVD versus BSC.** Scenario definition (labels): The percentage defines the responder rate and the Euro value defines the first year AVD costs in thousands (e.g. 25%; €100 means AVD response rate of 25% and the first year AVD costs of €100,000).

In Figure 5 it can be seen that the probability that AVD is cost-effective is 0.07 (worst case), 0.36 (base Case) and 0.75 (best case) when considering a WTP threshold of €40,000. Looking at a WTP threshold of €60,000 and €80,000 the probability that AVD is cost-effective is 0.22 and 0.31 (worst case), 0.53 and 0.63 (base case) and 0.85 and 0.89 (best case), respectively.

## Discussion

Early health economic evaluations differ from late-stage evaluations (e.g. those used at the time of reimbursement submission or as basis for health technology assessments) in that they are much more flexible and are designed to explore uncertainty. This means they typically operate on a less robust evidential basis than late-stage evaluations.

Also the presented early health economic evaluation of the future potential of next generation AVDs for treating blindness in Germany has to handle with three main uncertainty factors: the effect size of next generation AVDs, the costs of next generation AVDs and the WTP threshold that might be applied in RP patients. These key issues will be discussed in the following section in order to define important considerations for future health economic evaluations of AVDs (which are equivalents for the limitations of the current modelling approach) and to determine and conclude on the cost-effectiveness potential of next generation AVDs. The discussion will be completed by highlighting the current and the possible future clinical health economic evidence requirements, and thus the possible hurdles for obtaining statutory reimbursement coverage, for next generation AVDs in Germany.

### Effect size and efficacy and safety

As the pre-clinical investigations for the next generation AVDs are currently ongoing different efficacy scenarios have been simulated. To simulate the efficacy of AVDs it was assumed that the vision of a specific proportion of patients (responders) improves in a manner that they are no more categorized as blind (visual acuity >20/200) which was then transferred into a quality of life improvement. Thus the current model does not take into account a positive effect of minor vision improvements (e.g. in non-responders) which might underestimate the efficacy of AVDs. Once published clinical trial data on next generation AVDs is available it will be possible to apply an approach that takes into account the quality of life impact of smaller vision improvements in order to avoid an underestimation of the AVD efficacy.

The current modelling approach assumes that the vision improvement will be maintained over the patients’ lifetime. This procedure is based on the assumption that the AVD implants (as well as the external parts) will maintain its function over a lifetime period. Even in future health economic evaluations it will be difficult to eliminate this limitation as such long-term data on the AVD function will not be available at the time of marketing approval; such data may be available a long time after marketing if the post-marketing surveillance works well. In order to ensure at least a minimum functioning period, AVD manufacturers should think about guaranteeing the function of AVDs over a specific time period. Using such data it will be possible to simulate two scenarios: once assuming a lifetime function and the other assuming that the AVD will work only for the guaranteed function period. Simulating a guaranteed function period (shorter than lifetime) will increase the ICER estimates, as it was seen in the one-way sensitivity analyses on the observation period (10 years vs lifetime).

The current model does not take into account the possible impact of the safety profile of AVDs on the efficacy (quality of life) as there is currently no safety data available for next generation AVDs. In order to restore the vision in a manner that the patients’ vision improves above the blindness threshold the next generation AVDs need to enable a strongly increased resolution which will highly likely be linked to more adverse effects. This is based on the fact that achieving a higher resolution requires that more electrodes need to implanted and that these need to be set closer to each other which also affects the amount of electrical power that can be delivered safely to target neuronal tissue. Hence next generation AVDs will operate near the natural charge/density limits which might lead to more adverse effects [4,22]. Therefore future assessments based on clinical trial outcomes should take into account the influence of such adverse effects on the patients’ quality of life and should so enable to simulate the safety profiles’ impact on the QALY estimates.

Furthermore a future modelling approach should consider to base the cost-effectiveness outcomes not only on QALY estimates but to use additional clinical markers e.g. the visual acuity itself in order to express cost-effectiveness differently. The rationale for this procedure is that the QALY approach is seen critical by German authorities (especially by the IQWiG) and hence using clinical markers, to provide additional cost-effectiveness outcomes, will broaden the spectrum for argumentation. Outcomes such as vision years gained are commonly applied in visual impairment/blindness cost-effectiveness assessment and performing such analyses will hence enable a comparison of this cost-effectiveness outcome to other studies in the field.

### Pricing, healthcare costs and perspective

As there is currently no information available on the possible pricing of next generation AVDs, different pricing scenarios have been simulated using the price of currently available AVDs as basis. In order to present a wide range of possible pricing scenarios these scenarios take into account prices that are higher and lower than the current AVD costs. Although pricing scenarios assuming lower AVD prices has been simulated it is highly likely that the next generation AVDs will be more expensive that the current generation, as the growing competition in this market forces manufacturers to obtain the return of investment in a shorter period of time. Furthermore the anticipated incremental efficacy delivers a good argument for a price increase of next generation AVDs as such procedure reflects a kind of value-based pricing approach (additional money for an additional effect) that is already in use for pharmaceuticals in Germany.

Another important aspect of AVD costing is the estimation of annual follow-up costs that should reflect the costs of ongoing training and monitoring, the costs of possible device and software updates and the costs of possible adverse effects. In the presented assessment these costs were assumed at €1,500 which might conservatively overestimate the annual follow-up costs of next generation AVDs. Varying this estimate in one-way sensitivity analyses obtained that the amount of follow-up costs has a strong impact on the ICER and hence future assessments should perform the estimation of follow-up costs very accurately in order to avoid an over- or underestimation of the follow-up effort and subsequently of the ICER.

As requested by German health economic guidelines [15] a healthcare payer perspective was applied in order to estimate the cost-effectiveness of next generation AVDs. However, focusing on the healthcare payer perspective might underestimate the positive impact of a vision improvement. Lafuma et al. estimated the non-medical costs of visual impairment in four European countries including Germany and concluded that the “total non-medical costs associated with visual impairment are considerable” and that “hence the economics of visual impairment must be considered from a societal perspective” [23]. Taking this into account in combination with the issue, that next generation AVD prices will highly likely be located at the upper limited applied in our analyses, future health economic assessments should consider an additional investigation of the societal perspective, which would broaden the basis for the cost-effectiveness discussion on AVDs.

### Willingness to pay threshold and deviation of results

As mentioned previously there is no official WTP threshold per QALY gained in Germany. However, looking at the outcomes of ARMD cost-effectiveness assessments the efficiency frontier approach might justify a WTP of around €80,000 per QALY gained (please refer to the ‘Interpretation of Results’ section). In light of the official WTP thresholds in the United Kingdom that range between ≈ €24,000 and ≈ €36,000 (£20,000–£30,000) [24] this value seems to be far too high to be acceptable. However, there are other approaches e.g. that introduced by the WHO that connect the WTP to the economic capacity of a country [25]. According to this WHO approach an intervention is highly cost-effective if the ICER is less than the GDP per capita and cost-effective if the ICER is between one and three times the GDP per capita. Applying the German GDP per capita (€30,484 in 2013) [26] the WHO WTP threshold is ranging between ≈ €30,000 and ≈ €90,000 which is more in line with the WTP estimate based on the ARMD cost-effectiveness studies. As the WTP threshold cannot be predicted accurately different WTP thresholds have been applied in order to investigate the probability of whether AVD is a cost-effective treatment strategy in RP when compared to BSC. In these analyses it was obtained that there is a relatively high probability that next generation AVDs will be regarded as cost-effective intervention if the WTP threshold is located at €80,000 with declining probabilities if the WTP threshold is reduced.

Hence the cost-effectiveness of next generation AVDs will mainly depend on the acceptable WTP threshold that will be defined by German authorities on the basis of the efficacy frontier approach. As there are no RP specific cost-effectiveness analyses available it is highly likely that the ARMD cost-effectiveness thresholds will be applied which will result in a relatively high WTP threshold located around €80,000 per QALY gained. Irrespective of the discussions around the WTP threshold it needs to be considered that a reimbursement decision of AVDs, especially in the German setting, will not be limited to the outcomes of a cost-effectiveness assessment. Other, even more important decision criteria, such as the innovative nature of the AVD technology, the absence of effective therapy methods in RP (high unmet medical need) as well as the unprecedented therapy benefit (making the blind see again) will be taken into account in order to decide on the value and subsequently on the reimbursement of next generation AVDs.

### Current and future clinical and health economic evidence requirements

The German reimbursement requirements for medical devices are based on central regulating laws in the social code book V that state that in the inpatient care setting all (innovative) procedures are permitted with the reservation of prohibition (German: Verbotsvorbehalt) [27] and that in the outpatient care setting all (innovative) procedures are prohibited until they have been officially approved (German: Erlaubnisvorbehalt) [28]. This leads to the case that medical devices applied in the German inpatient setting require very limited clinical and health economic evidence whereas strong clinical and health economic evidence is required for medical devices applied in the German outpatient setting [29]. As AVDs are implanted in an inpatient setting the clinical and health economic evidence requirements are relatively low but it is highly recommend to summarize all available evidence (clinical & health economic) in order to support hospitals in the reimbursement fee negotiations with the healthcare payers.

However, there are recent political and scientific streams [30] that claim that the regulatory and reimbursement process of medical devices should be adapted comparable to the more restrictive regulations for pharmaceuticals, which might have a major impact on the evidence requirements for the inpatient sector. In case that the medical device regulation would be aligned with the process for pharmaceuticals, according to the act on the reform of the market for medical products (German: Arzneimittelmarkt-Neuordnungsgesetz; abbreviation: AMNOG) this would mean that a detailed clinical and health economic evidence reporting might be required to obtain reimbursement in Germany, even for medical devices applied in the inpatient setting.

Beside the possible changes of regulations, and a related possible increase of evidence requirements, there is another key issue that inpatient medical devices are facing, which is related to the kind of health economic assessments accepted by healthcare payers. The German healthcare payers are used to focus on a short term perspective due to their yearly budget viewpoint and as a consequence they consider short-term budget impact to be more important than long-term cost-effectiveness. This fact is also reflected in the current AMNOG regulations for pharmaceuticals that request only a short term budget impact analysis but no (long-term) cost-effectiveness assessment. This issue is of special importance for implantable medical devices, such as next generation AVDs, as a large part of the related costs are generated in the year of implantation. These high first year cost require that long term efficacy outcomes are taken into account in order to obtain a cost-effective relation between benefits and costs. Thus future regulations on health economic evidence requirements need to take into account these special characteristics of medical devices and should therefore set the focus on (long-term) cost-effectiveness and not on short-term budget impact.

## Conclusions

In conclusion the presented early cost-effectiveness evaluation has obtained that next generation AVDs have the potential to be a cost-effective therapy option in patients with RP in Germany. The innovative nature, the high unmet medical need and the expected unprecedented efficacy of next generation AVDs will highly likely lead to the case that even relatively high incremental cost-effectiveness ratios, that have been obtained when simulating various effect and pricing scenarios, will be regarded as acceptable from a German healthcare payer perspective.

## Abbreviations

AMNOG: Arzneimittelmarkt-Neuordnungsgesetz (English: act on the reform of the market for medical products)
ARMD: Age-related macular degeneration
AVD(s): Artificial vision device(s)
BSC: Best supportive care
IC: Incremental costs
ICER(s): Incremental cost-effectiveness ratio(s)
IE: Incremental effect
IQWiG: German institute for quality and efficacy in healthcare
RP: Retinitis pigmentosa
RR: Responder rate
SD: Standard deviation
QALY(s): Quality-adjusted life year(s)
WHO: World health organization
WTP: Willingness-to-pay
